# Two Cases of Crovalimab-Induced Platelet Recovery in Bone Marrow Failure-Associated Paroxysmal Nocturnal Hemoglobinuria (PNH)

**DOI:** 10.7759/cureus.84380

**Published:** 2025-05-19

**Authors:** Takashi Onaka, Yusuke Yamada, Kazunori Imada

**Affiliations:** 1 Hematology, Japanese Red Cross Osaka Hospital, Osaka, JPN

**Keywords:** anemia, bone marrow failure, crovalimab, paroxysmal nocturnal hemoglobinuria (pnh), thrombocytopenia

## Abstract

Paroxysmal nocturnal hemoglobinuria (PNH) is a rare, acquired clonal hematopoietic stem cell disorder caused by phosphatidylinositol N-acetylglucosaminyltransferase subunit A (PIG-A) gene mutations, leading to complement-mediated hemolysis, cytopenias, and thrombotic risk. While C5 inhibitors, such as eculizumab and ravulizumab, have transformed PNH management, optimal strategies for bone marrow failure-associated PNH remain unclear. We report two cases of bone marrow failure-associated PNH treated with crovalimab, focusing on its effects on hemolysis and blood cell recovery, particularly platelet counts within four weeks of crovalimab initiation. Case 1: A 79-year-old man presented with mild anemia, thrombocytopenia, elevated LDH, and impaired renal function. Crovalimab initiation resulted in rapid symptom improvement, decreased LDH, and a platelet increase. Case 2: A 22-year-old male athlete exhibited mild anemia, thrombocytopenia, and elevated LDH. Crovalimab resolved the symptoms, there were no hemolytic attacks, and the platelet count increased. Both patients demonstrated prompt improvement in hemolytic parameters and significant platelet recovery following crovalimab initiation, with no serious adverse events. These findings suggest that crovalimab not only attenuates hemolysis but may also improve platelet counts in bone marrow failure-associated PNH, highlighting its broader therapeutic potential and the need for further investigation in larger cohorts.

## Introduction

Paroxysmal nocturnal hemoglobinuria (PNH) is an acquired clonal disorder of hematopoiesis characterized by intravascular hemolysis and manifested by episodes of hemoglobinuria and life-threatening venous thromboses [1]. PNH is caused by mutations in the phosphatidylinositol N-acetylglucosaminyltransferase subunit A (PIG-A) gene of hematopoietic stem cells [2]. Treatment of PNH has made significant progress with the emergence of complement inhibitors, which have improved anemia by controlling hemolysis and reduced the risk of developing thrombosis [3]. Clinically, PNH is classified into three categories: classical PNH, bone marrow failure-associated PNH, and mixed type [4]. While the advent of complement inhibitors has significantly advanced the treatment of classical PNH in recent years, the optimal therapeutic strategy for bone marrow failure-associated PNH remains debatable. In the consensus statement for PNH described by Cançado et al., it is stated that complement inhibitors for bone marrow failure-associated PNH may benefit patients who present with high hemolytic burden [5]. Crovalimab is unique in that it has a longer biological half-life than other C5 inhibitors and can be administered by subcutaneous injection, although the efficacy of the novel complement inhibitor crovalimab in bone marrow failure-associated PNH is more unclear. We herein report two cases of bone marrow failure-associated PNH in which he administration of crovalimab not only improved hemolytic parameters, but also led to the recovery of other blood cell counts.

## Case presentation

Case 1

**Figure 1:**
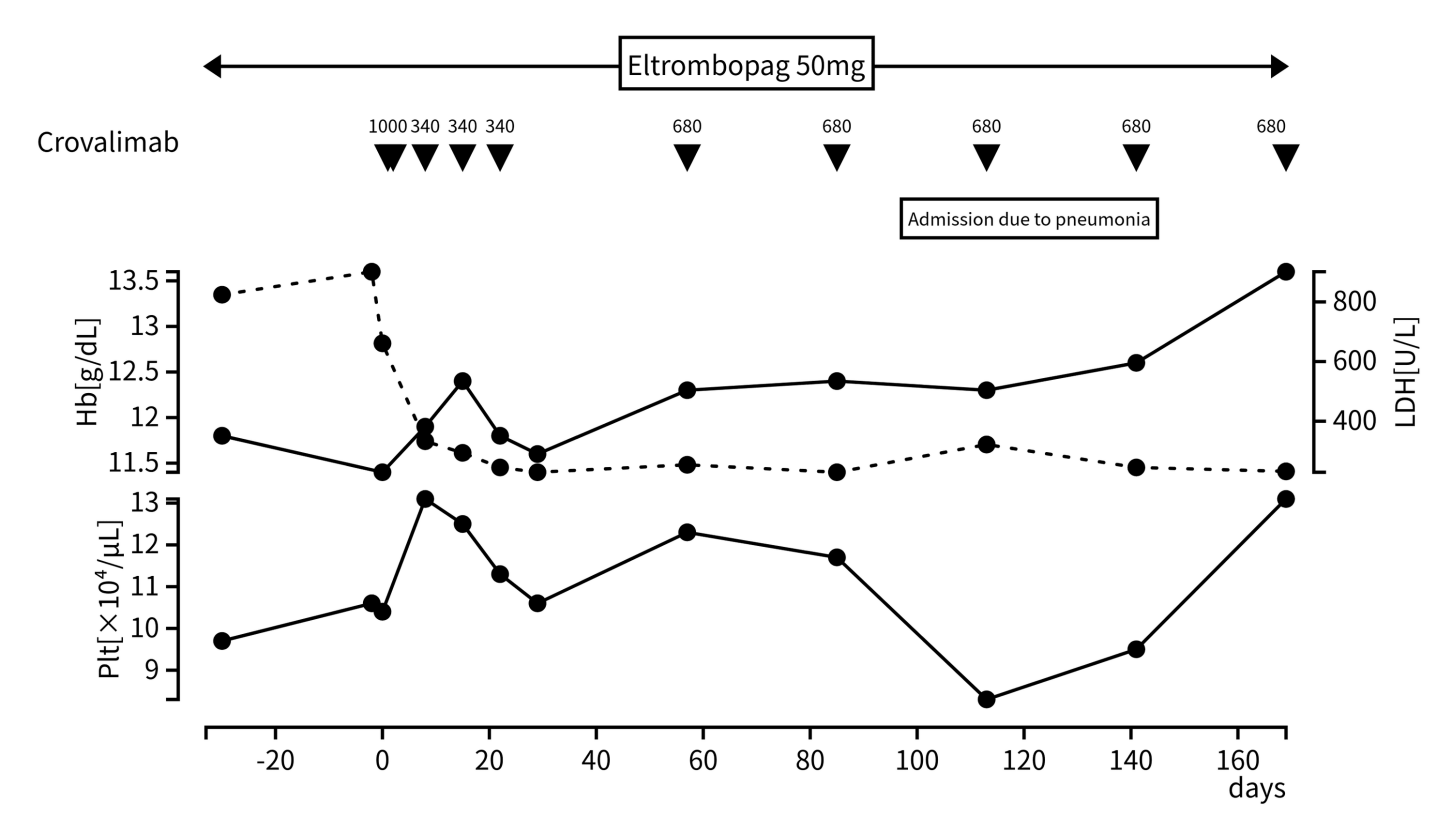
Case 1 clinical course When the patient was started on crovalimab, his LDH level slightly decreased, and hemolytic attacks improved. His platelet count also increased to 130,000/µL.

A 79-year-old man presented to our hospital with general fatigue. He also had a history of early morning hemoglobinuria. A blood examination revealed a slightly decreased hemoglobin level, an elevated reticulocyte count, lactate dehydrogenase (LDH) level, and bilirubin level. In addition, his creatinine level was elevated at 1.51 mg/dL, indicating impaired renal function. He was previously diagnosed with immune thrombocytopenia and started on oral eltrombopag 50 mg monotherapy in 2018. Although there was no improvement in his platelet levels, he did not receive a dose modification. Peripheral blood testing for CD55 and CD59 double-negative red cells was positive at 12.07%, confirming the diagnosis of PNH in April 2022. In addition, while the peripheral white blood cell count was within normal limits, the platelet count was reduced to 80,000/μL in spite of treatment with eltrombopag, leading to the diagnosis of bone marrow failure-associated PNH. The administration of crovalimab was initiated in September 2024 and quickly attenuated malaise; however, temporary headaches appeared at the time of introduction of crovalimab. His LDH level decreased markedly, and hemolytic attacks improved. Although transient thrombocytopenia was observed during admission due to pneumonia therapy, his platelet count also increased to 130,000/µL without any thromboembolism event (TE) (Figure 1).

Case 2

**Figure 2:**
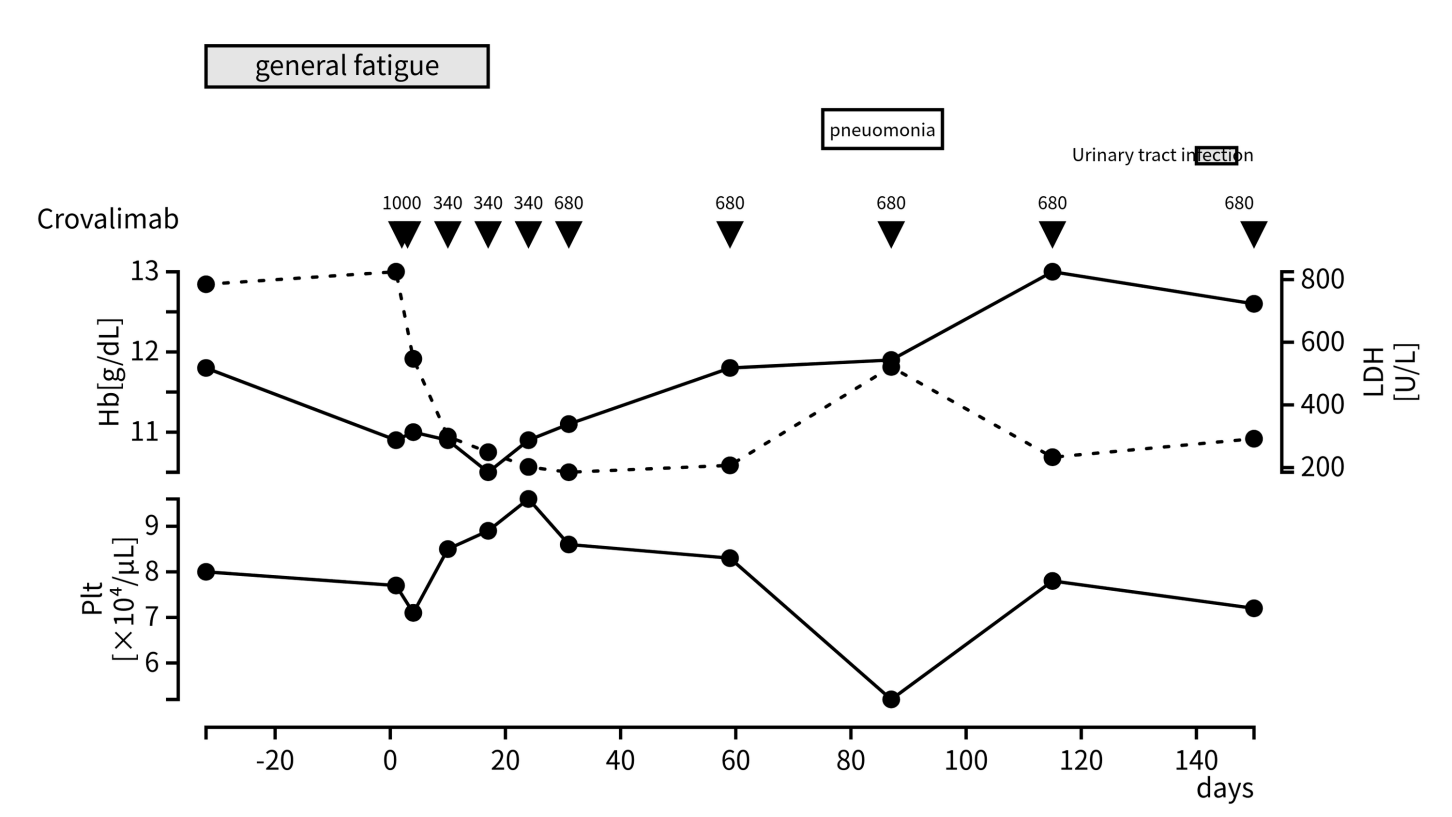
Case 2 clinical course When the patient was started on crovalimab, he was able to avoid hemolytic attacks, and his platelet count increased to 96,000/µL.

A previously healthy 22-year-old male competitive swimmer began experiencing training difficulties for the first time two years ago. He presented to a local clinic with morning hematuria. Thrombocytopenia and mild anemia were detected, and thus, he was referred to our facility. At presentation, his platelet count was 60,000/μL, his LDH level was elevated, and his hemoglobin level was 10 g/dL. Flow cytometry showed 17.42% double-negative red cells for CD55 and CD59, leading to a diagnosis of bone marrow failure-associated PNH in March 2023, which was followed up without treatment. The administration of crovalimab was initiated in October 2024 and quickly attenuated malaise with no side effects. Hemolytic attacks did not occur, and the patient was able to return to his previous training intensity. Although thrombocytopenia was observed several times during the infectious complications, his platelet count also slightly increased to 96,000/µL, with reduced bleeding risk and no TE. He had not received prior immunosuppressive therapy or thrombopoietin receptor agonists during the entire course (Figure 2).

## Discussion

We herein report two patients with bone marrow failure-associated PNH who were treated with crovalimab, which increased their platelet counts and attenuated hemolysis.

In addition to their efficacy in reducing intravascular hemolysis, C5 inhibitors have been associated with a reduction in thrombotic events, a major cause of morbidity and mortality in PNH patients [6]. However, their impact on platelet counts in PNH has been a subject of debate. Platelet counts increased in some cases with existing C5 inhibitors [7] but not in others [8].

Cohort studies also reported mixed findings. Kelly et al. examined platelet counts after treatment with eculizumab in 61 patients with PNH [9]. They found no significant change in platelet counts after eculizumab in their evaluation of 12 patients with reduced platelet counts. 

However, in the study by Gurnari et al., platelet counts were significantly higher in patients treated with eculizumab than in those not treated with eculizumab [10]. Socie et al. demonstrated that eculizumab, a C5 inhibitor, reduced complement-mediated platelet consumption in PNH patients [11]. Significant reductions in TE and the restoration of platelet counts were observed in thrombocytopenic PNH patients treated with eculizumab, with 36% achieving non-thrombocytopenic levels after 52 weeks.

These studies also showed that thrombocytopenia in PNH was closely associated with an elevated risk of TE, with thrombocytopenic patients being more likely to develop TE than those with normal platelet counts. Platelet activation, driven by terminal complement deposition, has been suggested to contribute to chronic platelet consumption and a prothrombotic state. These findings support the hypothesis that terminal complement activation plays a role in both platelet activation and consumption, thereby promoting thrombocytopenia and increasing the risk of thrombosis in PNH patients. Although controversial, the present results might indicate the potential benefits of treatments with C5 inhibitors in other aspects of blood cell recovery. Crovalimab's sustained C5 inhibition (t₁_/_₂=53.1 days [12] vs. eculizumab's 187.7 hours [13]) might lead to platelet recovery and suggest the beneficial role of crovalimab in treating bone marrow failure-associated PNH. The present cases both had intermediate-stage bone marrow failure-associated PNH, with only mildly decreased platelet counts. The disease severity and relatively early administration of crovalimab, within three years of the diagnosis of PNH, may have contributed to the therapeutic effects of crovalimab.

## Conclusions

In conclusion, our cases might be suggestive of the potential of crovalimab to not only control hemolysis but also contribute to platelet count recovery in patients with bone marrow failure-associated PNH. Crovalimab may have broader hematologic benefits beyond the control of hemolysis based on the rapid and sustained improvement observed in both patients. Therefore, crovalimab may have an impact on reducing complement-mediated platelet consumption and improving the bone marrow microenvironment. Since there have been no other findings to show that crovalimab, a novel subcutaneous C5 inhibitor, improves platelet counts, a registry study of crovalimab-treated PNH patients with bone marrow failure is warranted.
